# Anti-CGRP monoclonal antibodies for chronic migraine with medication-overuse headache: a conservative meta-analysis

**DOI:** 10.1055/s-0046-1817018

**Published:** 2026-02-27

**Authors:** Luana Miyahira Makita, Gabriela das Graças dos Santos Carolino, Yuri Gubitose de Souza, Heloísa Carneiro Brito, Mariana Oliveira, Giovana Schlichta Adriano Kojima, Milena Ramos Tomé, Júlia dos Santos Monteiro, Yasmin Bastos Faller, Elcio Juliato Piovesan, Mario Fernando Prieto Peres

**Affiliations:** 1 Universidade Federal do Paraná, Setor de Ciências da Saúde, Departamento de Medicina, Curitiba PR, Brazil.; 2 Faculdade de Ciências Médicas e da Saúde de Juiz de Fora, Departamento de Medicina, Juiz de Fora MG, Brazil.; 3 Faculdade de Medicina do ABC, Departamento de Medicina, Santo André SP, Brazil.; 4 Universidade Federal da Paraíba, Departamento de Medicina, João Pessoa PB, Brazil.; 5 Faculdade de Ensino Superior da Amazônia Reunida, Departamento de Medicina, Redenção PA, Brazil.; 6 Universidade Federal de Campina Grande, Cajazeiras PB, Brazil.; 7 Universidade de Pernambuco, Recife PE, Brazil.; 8 Universidad de Buenos Aires, Departamento de Medicina, Buenos Aires, Argentina.; 9 Universidade Federal do Paraná, Departamento de Clínica Médica, Disciplina de Neurologia, Curitiba PR, Brazil.; 10 Hospital Israelita Albert Einstein, Departamento de Neurologia, São Paulo SP, Brazil.; 11 Universidade de São Paulo, Faculdade de Medicina, Hospital das Clínicas, Departamento de Psiquiatria, São Paulo SP, Brazil.

**Keywords:** Migraine Disorders, Calcitonin Gene-Related Peptide, Antibodies, Monoclonal, Headache Disorders, Secondary, Disease Prevention

## Abstract

**Background:**

Chronic migraine (CM) is often complicated by medication-overuse headache (MOH), worsening disability. Although withdrawal of overused medications is recommended, adherence is poor and relapse is frequent. Monoclonal antibodies (mAbs) targeting the calcitonin gene-related peptide (CGRP) pathway may offer an effective preventive option without requiring discontinuation, but current evidence is limited by few and heterogeneous randomized controlled trials (RCTs).

**Objective:**

To systematically assess the efficacy and safety of anti-CGRP mAbs in treating CM with MOH (CM + MOH) under a conservative approach.

**Methods:**

The PubMed, Embase, and Cochrane Central databases were searched for RCTs comparing anti-CGRP mAbs with placebo in adults with CM + MOH. The primary outcome was the mean change in monthly migraine days (MMDs) at 3 months. The secondary outcomes included acute medication use, disability, drug overuse resolution, response rate, and adverse events (AEs). Random-effects models with Sidik-Jonkman estimator and Knapp-Hartung adjustments pooled effect sizes.

**Results:**

We included seven RCTs, totalling 3,094 patients. Anti-CGRP mAbs significantly reduced MMDs (mean difference [MD] = -0.35; 95%CI: -0.43 to -0.26) and acute medication use (MD = -0.35; 95%CI: -0.51 to -0.19) compared with placebo. Higher rates, of ≥ 50%, of response (risk ratio [RR] = 1.94; 95%CI: 1.60–2.34) and drug overuse resolution (RR = 1.38; 95%CI: 1.04–1.83) were observed, with no significant increase in AEs (RR = 1.09; 95%CI: 0.85–1.40).

**Conclusion:**

Anti-CGRP mAbs were effective and well tolerated in CM + MOH, representing a viable alternative, especially for patients unable to discontinue acute medications. Further research should assess long-term outcomes and subgroup effects.

## INTRODUCTION

Migraine is a highly-prevalent and debilitating neurological disorder, affecting approximately 1.16 billion people worldwide. 1 Despite its impact, migraine remains underdiagnosed, undertreated, and stigmatized, leading to gaps in clinical care and research. 2 3 Chronic migraine (CM) imposes an even greater burden and is defined by the International Classification of Headache Disorders, 3rd edition (ICHD-3), as headaches occurring on ≥ 15 days per month for ≥ 3 months a year, with at least 8 days featuring migraine characteristics. 4 Chronic migraine is frequently associated with the overuse of acute medications, including analgesics, triptans, and opioids. Such overuse can result in increased pain intensity and frequency, ultimately leading to medication-overuse headache (MOH), a secondary headache disorder. 5 The diagnosis of MOH is established in individuals with a preexisting primary headache disorder who experience headaches on ≥ 15 days per month, resulting from the regular overuse of acute or symptomatic headache drugs for > 3 three months. 4 The coexistence of CM and MOH (CM + MOH) complicates treatment, as these patients experience more severe disease courses and often exhibit resistance to standard therapies. 5

The conventional treatment for MOH generally involves early withdrawal of overused medications, with the simultaneous or subsequent initiation of preventive therapy. 6 However, this removal-centered approach to treatment has been criticized due to implementation challenges in the clinical practice, high rates of treatment failure, the potential for patient self-blame, and frequent relapses. 2 7 In a qualitative study, 8 interviews revealed that MOH participants regarded acute medication as indispensable, fearing disruptions in daily life without it.

Anti-calcitonin gene-related peptide (CGRP) pathway monoclonal antibodies (mAbs) are now considered first-line options for migraine prophylaxis. 9 Recent randomized controlled trials 10 11 (RCTs) have suggested these newly-developed therapies may be beneficial in treating patients with CM + MOH, even without strict withdrawal of medication. However, evidence remains limited. A previous meta-analysis 12 addressed this topic but only included four RCTs. Since then, more studies have been published, resulting in a substantial increase in the available sample size (> 50%) and outcomes. Therefore, we conducted an updated systematic review and meta-analysis to assess the efficacy and safety of mAbs targeting the CGRP ligand or its receptor, anti-CGRP (receptor) mAbs, in patients with CM + MOH, and to explore the effects of the mechanism of action, treatment duration, and triptan use on clinical outcomes. Our analysis will adopt a conservative statistical method to reduce the risk of overestimating precision. This approach will enable us to determine whether the previously-observed benefits remain significant even under a more stringent analytical framework.

## METHODS

The current systematic review and meta-analysis was registered in the International Prospective Register of Systematic Reviews (PROSPERO, under protocol CRD42024607947). The study was designed and conducted in accordance with the 2020 guidelines of the Preferred Reporting Items for Systematic Reviews and Meta-Analysis 13 (PRISMA) statement and the *Cochrane Handbook for Systematic Reviews of Interventions* . 14

### Study eligibility

Inclusion in the meta-analysis was restricted to studies meeting all of the following criteria:

- RCTs and their secondary analyses;
- studies evaluating adults diagnosed with CM + MOH according to the ICHD-3; 4
- patients undergoing preventive treatment with mAbs targeting the CGRP ligand (fremanezumab, galcanezumab, or eptinezumab) or its receptor (erenumab);
- comparisons with placebo.

There were no restrictions on drug dosages. Studies were excluded if they:

- were non-placebo-controlled, observational, opinion articles, conference abstracts, brief communications, or similar types of publications;
- did not report relevant outcomes;
- involved overlapping populations; or
- were published in languages other than English.

### Search strategy

We systematically searched PubMed, Embase, and Cochrane Central Register of Controlled Trials from inception to October 2024. The detailed search strategy is presented in **Supplementary Material** (available at https://www.arquivosdeneuropsiquiatria.org/wp-content/uploads/2025/12/ANP-2025.0272-Supplementary-Material.docx ) **Table S1** . The references from all included studies, previous systematic reviews, and meta-analyses were also searched manually for any additional data. We did not perform searches in gray literature.

### Endpoints and subgroup analysis

The primary outcome was the mean change from baseline in the number of monthly migraine days (MMDs). The secondary outcomes included:

- mean change from baseline in the monthly acute headache medication days (AHMDs);
- transition of MOH status to non-MOH;
- mean change from baseline in the score on the 6-Item Headache Impact Test (HIT-6);
- ≥ 50% responder rates in MMDs; and
- the incidence of treatment-emergent adverse events (TEAEs).

A meta-analysis was performed for the outcomes whose data were provided from at least three studies. We performed subgroup analyses for the outcomes of interest according to the mAb type, treatment duration, and triptan overuse status. The predefined subanalysis stratified to different time points (that is, at month 6) was not possible due to the insufficient data.

### Data extraction

Title, abstract, and full-text screening were performed independently by two investigators (LMM and GSAK). Any discrepancies between the assessors were resolved by discussion until a consensus was reached. Data was extracted by four investigators (LMM, HCB, MO, and MRT) using a spreadsheet with predetermined data fields. Additionally, we collected data on study design, number of participants, and population characteristics. For standardization, outcomes were extracted 3 months after treatment initiation whenever possible. When this timeframe was unavailable, the discrepancy was noted in the forest plot legends (*) to highlight potential disparities.

If necessary, specific sources were used to combine mean ± standard deviation (SD) values from multiple groups, 15 calculate the SD by imputing the CI values in one group, 16 and extract the data when provided as a figure. 17 To calculate the change in mean ± SD from baseline by providing the values before and after an event, 14 a correlation coefficient was assumed to be 0 in a conservative approach, to minimize the risk of false positives. If necessary, SDs were calculated based on available information (SD = standard error [SE] × √N). This was important since many trials reported the pooled effect in least squares mean and SE format.

### Quality assessment

The methodological quality of all included studies was evaluated independently by two authors (JSM and YBF) using the revised Cochrane risk of bias tool for randomized trials, version 2.0 (RoB 2). 18 Disagreements were resolved by consensus after discussing reasons for discrepancy with a third researcher (LMM).

Publication bias was assessed by visual inspection of funnel plot asymmetry. A funnel plot was generated by plotting the effect estimates against their corresponding SEs for the included studies. We did not perform formal statistical testing for publication bias due to the limited number of studies (n < 10). 14

### Data analysis

For the continuous outcomes, we conducted a meta-analysis using the mean difference (MD) ± SD, accompanied by 95%CIs. For the binary outcomes, proportions of patients were analyzed using risk ratios (RRs). Study weights were assigned using the generic inverse-variance method for the continuous outcomes and the Mantel-Haenszel method for the binary outcomes. A random-effects model with the Sidik-Jonkman estimator and Knapp-Hartung adjustments was used to ensure a conservative approach, accounting for the small sample size and expected heterogeneity from post-hoc subgroup analyses. This combination is particularly suited to reduce the risk of type-I error in meta-analyses with limited data or moderate-to-high variability. 19 The significance level was set at 5%. Heterogeneity was assessed using prediction intervals (PIs) for the binary outcomes and I ^2^ statistics for the continuous data, with I ^2^ > 30% indicating substantial heterogeneity. 14 Leave-one-out sensitivity analyses were planned for major continuous outcomes in which I ^2^ exceeded 30%. The statistical analysis was performed using the R (R Foundation for Statistical Computing) software, versions 4.2.1 and 4.4.0, through R Studio.

## RESULTS

### Studies included and baseline characteristics

**Figure 1:**
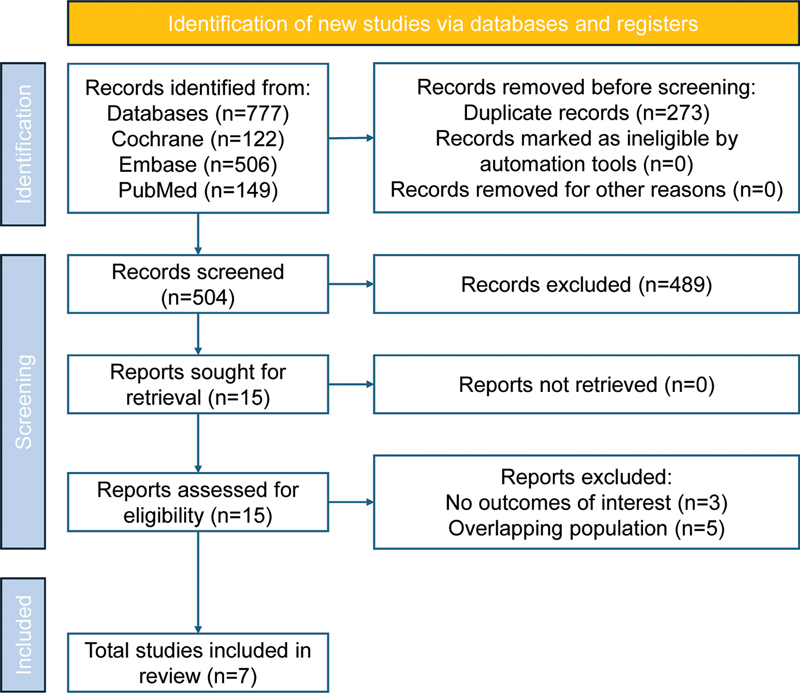
Preferred Reporting Items for Systematic reviews and Meta-Analyses (PRISMA) flow diagram of study screening and selection.

As illustrated in Figure 1 , a comprehensive literature search initially identified 777 articles. After removing duplicates and excluding unrelated articles based on title and abstract screening, 15 studies were selected for full-text review. The main reasons for withdrawal were the absence of outcomes of interest and overlapping populations ( **Supplementary Material Table S2** ). We included seven RCTs involving 3,094 patients with CM + MOH, 2,065 (66.7%) of whom received anti-CGRP (receptor) mAbs for the treatment of migraine. 10 11 20 21 22 23 24 Detailed characteristics of these studies are presented in Table 1 .

**Table 1:** Detailed characteristics of the studies included in the meta-analysis

												Study design duration, weeks				
Study (year)	Intervention	Dosage	Study design allocation	Age, mean + SD or median (IQR)	Total	Female sex (%)	BMI in kg/m ^2^ (mean ± SD)	Exclusion of patients with opioid overuse	Monthly migraine days, mean ± SD	Patients using triptans (%)	Acute medication headache use, mean ± SD	Screening	Baseline	Double-blind treatment	Open-label treatment	Follow-up
** Silberstein et al. 21 (2020)• **	Fremanezumab	Monthly (675/225/225 mg) and quarterly (675 mg/PBO/PBO)	Parallel (1:1:1)	44.80 ± 11.10	587	524 (89.3)	26.3 ± 5.1	Yes	17.3 ± 4.8	407 (69.3)	18.2 ± 4.3	−	4	12	−	−
** Imai et al. 22** **(2023)•**	Fremanezumab	Monthly (675/225/225 mg) and quarterly (675 mg/PBO/PBO)	Parallel (1:1:1)	43.83 ± 9.63	320	272 (85.0)	22.7 ± 3.6	No✞	16.6 ± 4.8	308 (96.3)	17.2 ± 4.7	4	−	12	−	−
** Diener et al. 20** **(2021)•**	Eptinezumab	100 mg and 300mg	Parallel (1:1:1)	41.4 ± 10.78	431	376 (87.2)	26.6 ± 5.21	Yes	16.7 ± 4.64	328 (76.1)	16.4 ± 6.4	4	−	24	−	8
** Dodick et al. 23** **(2021)•**	Galcanezumab	120mg and 240mg	Parallel (2:1:1)	43.5 ± 11.3	708	591 (83.5)	NA	Yes	20.0 ± 4.0	385 (54.4)	11.0 ± 2.0	1-6	4	12	36	16
** Yu et al. 10** **(2023)**	Eptinezumab	100 mg	Parallel (1:1)	43.5 (35–52)	190	148 (77.9)	NA	No	19.6 ± 3.7	NA	19.1 ± 4.6	4	−	12	12	8
** Tepper et al. 11** **(2019)•**	Erenumab	70mg and 140mg	Parallel (3:2:2)	43.4 ± 10.8	274	233 (85.0)	NA	Yes	19.0 ± 4.5	NA	13.1 ± 7.0	3	4	12	52	12
** Tepper et al. 24** **(2024)**	Erenumab	70mg and 140mg	Parallel (1:1:1)	43.7 ± 12.1	584	482 (82.5)	25.4 ± 5.2	Yes	18.8 ± 4.6	400 (68.5)	18.9 ± 4.2	3	4	24	28	−

Abbreviations: PBO, placebo; BMI, body mass index; IQR, interquartile range; SD, standard deviation; NA, not applicable.

Notes: Fremanezumab was administered in monthly doses of 225 mg or quarterly doses of 675 mg. **•** Data from the subpopulation with chronic migraine with medication overuse headache (MOH + CM) were obtained from secondary analyses of the randomized controlled trial. ✞As the study by Imai et al. 22 did not collect information to enable the subdivision of patients according to the types of overused medication, certain restrictions on the significance of clinical diagnosis and treatment must be acknowledged. However, at least in relation to opioids, overuse is considered very rare in Japan, given that opioids and barbiturates are not contained in over-the-counter combination analgesics and opioids cannot be prescribed for headache without terminal cancer pain.

### Primary outcome

**Figure 2:**
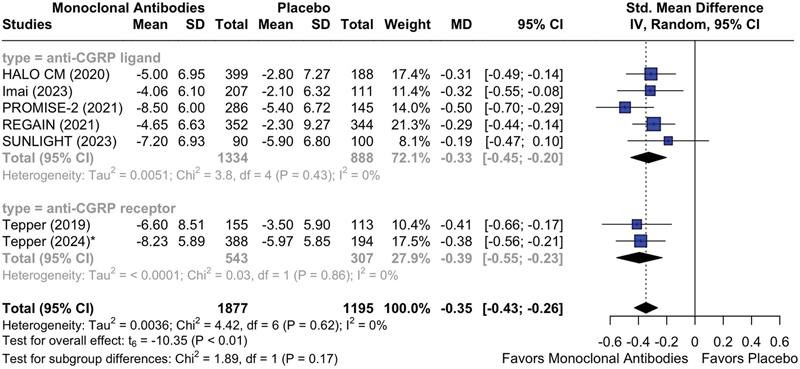
Abbreviations: MMDs, monthly migraine days; CGRP, calcitonin gene-related peptide; mAbs, monoclonal antibodies. Note: *Outcome provided at month 6. Mean reduction from baseline in MMDs at month 3 in patients treated with anti-CGRP (receptor) mAbs versus placebo, stratifying by mAb mechanism of action.

A total of seven studies (n = 3,072) evaluated the mean change from baseline in the MMDs at month 3 in patients with CM + MOH treated with anti-CGRP (receptor) mAbs. Pooled effects showed that, in comparison with placebo, mAbs were associated with statistically significant reduced migraine frequency (MD = -0.35; 95%CI: -0.43 to -0.26; I ^2^ = 0%; *p* < 0.05). A subgroup analysis based on the mechanism of action of mAbs demonstrated an MD of -0.33 (95%CI: -0.45 to -0.20; I ^2^ = 0%; *p* < 0.05) for mAbs that antagonize with the CGRP ligand, and an MD of -0.39 (95%CI: -0.55 to -0.23; I ^2^ = 0%; *p* < 0.05) for mAbs that antagonize with the receptor of CGRP ( Figure 2 ).

### Secondary outcomes

**Figure 3:**
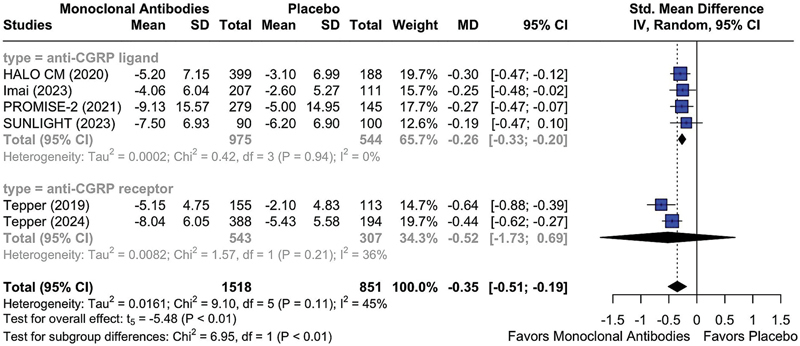
Abbreviations: AHMDs, acute headache medication days; CGRP, calcitonin gene-related peptide; mAbs, monoclonal antibodies. Mean reduction from baseline in AHMDs at month 3 in patients treated with anti-CGRP (receptor) mAbs versus placebo, stratifying by mAb mechanism of action.

The CGRP pathway antagonists demonstrated significantly greater efficacy than placebo in reducing the number of AHMDs at month 3 (MD = -0.35; 95%CI: -0.51 to -0.19; I ^2^ = 45%; *p* < 0.05) ( Figure 3 ). A subgroup analysis regarding the mechanism of action showed a significant reduction in AHMDs among patients receiving anti-CGRP ligand mAbs (MD = -0.26; 95%CI: -0.33 to -0.20; I ^2^ = 0%; *p* < 0.05), whereas no significant effect was observed for anti-CGRP receptor mAbs (MD = -0.52; 95%CI: -1.73–0.69; I ^2^ = 36%; *p* > 0.05) ( Figure 3 ), likely reflecting the substantial heterogeneity between the studies conducted by Tepper et al. in 2019 11 and by Tepper et al. in 2024, 24 as well as the limited number of available studies, which contributed to the wide CI. The sensitivity analysis indicated a reduction in heterogeneity (I ^2^ = 0%), with a corresponding narrowing of the confidence interval upon the exclusion of the study by Tepper et al. 11 ( **Supplementary Material Figure S1** ).

**Figure 4:**
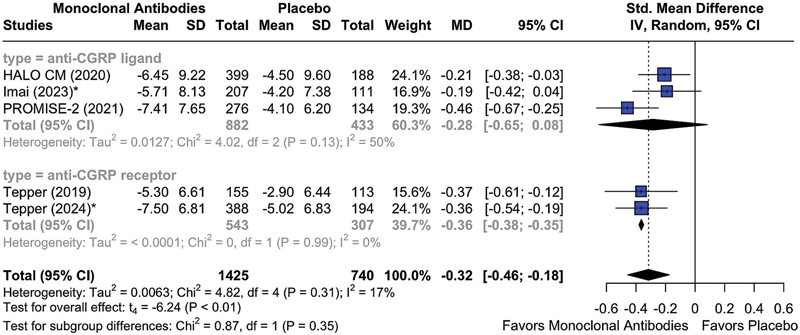
Abbreviations: HIT-6, 6-Item Headache Impact Test; CGRP, calcitonin gene-related peptide; mAbs, monoclonal antibodies. Note: *Outcome provided at month 5/6. Mean reduction from baseline in HIT-6 score at month 3 in patients treated with anti-CGRP (receptor) mAbs versus placebo, stratifying by mAb mechanism of action.

Regarding disability, the mean change in the HIT-6 scores at month 3 favored the mAb group over placebo (MD = -0.32; 95%CI: -0.46 to -0.18; I ^2^ = 17%; *p* < 0.05) ( Figure 4 ). Mechanism-based stratifications showed a significant benefit for receptor-targeting agents (MD = -0.36; 95%CI: -0.38 to -0.35; I ^2^ = 0%; *p* < 0.05), while neuropeptide-targeting antibodies did not yield a statistically significant effect (MD = -0.28; 95%CI: -0.65–0.08; I ^2^ = 50%; *p* > 0.05).

A ≥ 50% reduction in MMDs at month 3 was significantly more frequent among patients treated with mAbs compared to placebo (RR = 1.94; 95%CI: 1.60–2.34; *p* < 0.05), with PIs ranging from 1.21 to 3.11 ( **Supplementary Material Figure S2** ). No significant difference was observed in TEAEs between the intervention and control groups (RR = 1.09; 95%CI: 0.85–1.40; *p* > 0.05), with PIs ranging from 0.57 to 2.06 ( **Supplementary Material Figure S3** ).

**Figure 5:**
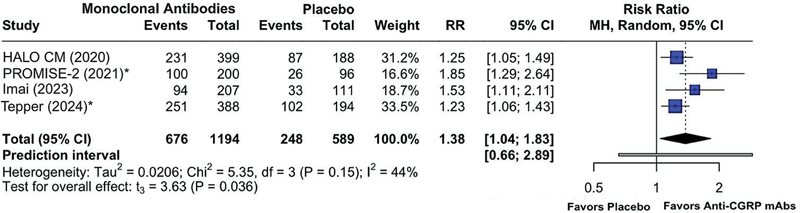
Abbreviations: CGRP, calcitonin gene-related peptide; mAbs, monoclonal antibodies; MOH, medication-overuse headache. Note: *Outcome provided at month 6. Risk ratio of the proportion of patients treated with anti-CGRP (receptor) mAbs versus placebo reverting from a MOH status to a non-MOH status at month 3.

The reversion of MOH to non-MOH status was reported in four studies (n = 1,783), demonstrating a significant benefit of anti-CGRP (receptor) mAbs over placebo (RR = 1.38; 95%CI: 1.04–1.83; *p* < 0.05), with PIs ranging from 0.66 to 2.89 ( Figure 5 ). In a subgroup of patients with triptan overuse, the RR increased to 1.63 (95%CI: 1.08–2.45; *p* < 0.05; PI: 0.23–11.62) ( **Supplementary Material Figure S4** ).

### Quality assessment

The individual bias assessment of the RCTs included is summarized in **Supplementary Material Figure S5** . The analysis revealed a predominantly-low risk of bias across all studies included, though a few studies were rated as presenting “some concerns” due to specific methodological limitations, primarily related to multiple analyses within a single trial and issues associated with deviations from intended interventions (Domain 2). There was no study with high risk of bias in any category. We also visually inspected the funnel plot and found no obvious signs of asymmetry ( **Supplementary Material Figure S6** )

## DISCUSSION

In the current systematic review and meta-analysis involving seven RCTs and 3,094 patients, we aimed to evaluate the efficacy and safety profile of the anti-CGRP (receptor) mAbs in preventing migraine in patients with CM + MOH. The pooled results demonstrated:

- a significant reduction in mean MMDs with mAbs versus placebo;
- a decrease in AHMDs with anti-CGRP mAbs, though not with receptor-targeting agents only;
- greater improvements in the HIT-6 score;
- higher ≥ 50% MMD response rates;
- comparable rates of TEAEs; and
- increased resolution of MOH, especially in triptan-overuse scenarios.

These findings are supported by a conservative analytical framework that reduces the risk of overstating statistical significance by appropriately scaling *p* -values and CIs. While this cautious methodology may yield smaller effect sizes and wider CIs, it enhances the credibility of the evidence, providing a robust foundation for clinical and policy decisions.

Previous meta-analyses have examined this topic. Sirilertmekasakul et al. 25 reported an RR of 1.44 for reversion to non-overuse status with anti-CGRP pathway mAbs in patients with episodic migraine or CM and MOH. Giri et al. 12 found a mean reduction of 2.68 MMDs in patients with CM + MOH using the DerSimonian–Laird method (n = 2,000; four studies). The employment of the Sidik-Jonkman estimator and Knapp-Hartung adjustments, as performed in the present study, has been shown 26 to provide more accurate error rates than the traditional DerSimonian-Laird method, especially in contexts of heterogeneity or limited number of studies. While previous work has demonstrated significant reductions in migraine frequency and drug overuse, the current meta-analysis confirms the robustness of these effects by accounting for new studies and for methodological limitations, thereby strengthening the reliability of the findings. Moreover, the present analyses go further, by evaluating quality of life measures, offering a clinical perspective on the significance of the results rather than focusing solely on statistical significance.

Providing effective migraine management can prevent attacks and interrupt the MOH cycle. 27 A real-world study 28 has shown that MOH patients treated preventively with erenumab, fremanezumab, or galcanezumab, without prior drug withdrawal, maintained reduced headache frequency and analgesic use, with low relapse rates after up to 1 year. The PREVAIL study reported significant long-term benefits of eptinezumab over 2 years, with 86.1% of the patients describing a “much improved” or “very much improved” status. 29 The current meta-analysis observed a slight difference between mAbs targeting the CGRP ligand versus the receptor, though both types of medications were statistically effective. 11 24 This may indicate a potentially-higher response to erenumab. However, limited data restrict definitive conclusions.

The pathophysiology of MOH is not fully understood and encompasses complex mechanisms involving headache-specific pain pathways, pharmacodynamic effects, and genetic predisposition. 30 Imaging studies 31 have revealed functional, morphological, and metabolic changes in the pain and addiction network in MOH, with normalization of pain regions after overuse resolution being common, but persistence of addiction. The recognized risk factors for the development of MOH comprise demographic, lifestyle, and drug-related factors, as well as self-reported health conditions. 30 Moreover, potential genetic risk factors, such as angiotensin-converting enzyme and brain-derived neurotrophic factor polymorphisms, have been demonstrated to influence dependence-behavior pathways. 32 33

Although anti-CGRP pathway mAbs have demonstrated preventive efficacy without the need for abrupt discontinuation of symptomatic drugs, it remains important to recognize that no universal approach applies to all MOH patients. In select cases, particularly those with complex headache profiles or longstanding overuse, a withdrawal plan may be clinically indicated. 34 Expert consensus 6 35 generally favors abrupt withdrawal, except in opioid overuse. Optimal treatment requires a multidimensional strategy that incorporates individual factors such as headache history, attack severity, comorbidities, and patterns of acute and preventive drug use. 36 Triptans, simple and combination analgesics, and opioids are most commonly implicated in MOH, 30 with triptan overuse being associated with a higher likelihood of migraine diagnosis, potentially due to increased medical attention. 37 In the present meta-analysis, triptan overuse ranged from 54.4 to 96.3%. Sex differences in the literature include shorter chronification latency in men and more severe phenotypes in women. 38 These results highlight the need for personalized strategies that address the unique characteristics of each migraine patient, going beyond exclusive reliance on pharmacological innovation.

Dependency-like behavior is most commonly seen in MOH patients who overuse opioids, which consequently predisposes to higher relapse rates when compared to other drugs. 39 In the current meta-analysis, most of the included RCTs excluded patients with opioid-overuse headache, which can affect the generalizability of the findings. Although opioids are frequently prescribed for the acute treatment of migraine in varied clinical settings, their use and real indication vary among countries and guidelines. 35 Healthcare professionals should be trained to identify beforehand patients at risk of MOH to prevent its development. 27

Finally, a crucial part of MOH treatment consists in education of the general population. Primary care plays a vital role in supporting patient autonomy to enable people with MOH to manage their own health and wellness. Kristoffersen et al. 40 evaluated the impact of advice on MOH patients and found that the percentage of patients no longer fulfilling the overuse criteria at the long-term follow-up was of approximately 70%, with a low relapse rate, of 8.3%. Providing educational materials and informing patients about the link between frequent pain-medication use and the progression from episodic to chronic headaches is an important step to prevent this condition.

### Limitations

The present study has several limitations. First, the use of the Sidik-Jonkman estimator with Knapp-Hartung adjustments may reduce statistical power, potentially classifying clinically-relevant effects as non-significant. Second, the inclusion of post-hoc analyses without prespecified hypotheses increases the risk of type-I error; however, our conservative approach helps mitigate this. All trials included were industry-sponsored, introducing potential funding bias, which we addressed through risk of bias assessments and publication bias analyses; however, residual bias cannot be excluded. Third, relapse rates could not be comprehensively analyzed due to limited reporting, underscoring the need for future studies to prioritize this outcome. Variability in patient education and potential behavioral changes at enrollment may also have influenced adherence and treatment response. Lastly, the exclusion of gray literature and non-English studies may have introduced publication bias, though none was evident in the funnel plot analysis. However, given the limited number of studies, the presence of publication bias cannot be ruled out and should be considered when interpreting the findings.

In conclusion, the current systematic review and meta-analysis demonstrated the efficacy and safety of prophylactic treatment with anti-CGRP (receptor) mAbs in patients with CM + MOH, significantly improving clinical outcomes even within a conservative analytical framework. These agents represent a valuable alternative to withdrawal-centered approaches, particularly for individuals initially unable or unwilling to discontinue acute medications. Further high-quality RCTs with rigorous monitoring are warranted to establish their long-term efficacy, especially in high-risk populations.
